# Differences and similarities of postprandial lipemia in rodents and humans

**DOI:** 10.1186/1476-511X-10-86

**Published:** 2011-05-23

**Authors:** Natalia B Panzoldo, Aline Urban, Eliane S Parra, Rogério Oliveira, Vanessa S Zago, Lívia R da Silva, Eliana C de Faria

**Affiliations:** 1 Department of Clinical Pathology, Lipid Laboratory and Center for Medicine and Experimental Surgery, Faculty of Medical Sciences (Rua Tessália Vieira de Camargo), University of Campinas, Campinas, (zip code 13084-971), Brazil; 2 Department of Statistics, Mathematics and Statistics Institute (Rua do Matão, 1010), University of São Paulo, São Paulo (zip code 05311-970), Brazil

**Keywords:** oral fat tolerance test, triglyceride-rich lipoproteins, remnant-like particle

## Abstract

**Background:**

The rat has been a mainstay of physiological and metabolic research, and more recently mice. This study aimed at characterizing the postprandial triglyceride profile of two members of the Muridae family: the Wistar rats (Rattus norvegicus albinus) and C57BL/6 mice (Mus musculus) plus comparing them to the profile obtained in humans.

**Methods:**

Thirty-one male and twelve female Wistar rats, ten C57BL/6 male and nine female mice received a liquid meal containing fat (17%), protein (4%) and carbohydrates (4%), providing 2 g fat/Kg. Thirty-one men and twenty-nine women received a standardized liquid meal containing fat (25%), dextromaltose (55%), protein (14%), and vitamins and minerals (6%), and providing 40 g of fat per square meter of body surface. Serial blood samples were collected at 2, 4, 6, 8 and 10 h after the ingestion in rats, at 1, 2, 3, 4, 5 and 6 h in mice and in humans at 2, 4, 6 and 8 h. Wilcoxon and Mann-Whitney tests were used.

**Results/Discussion:**

The triglyceride responses were evaluated after the oral fat loads. Fasting and postprandial triglyceridemia were determined sequentially in blood sample. AUC, AUIC, AR, RR and late peaks were determined.

**Conclusions:**

Rats are prone to respond in a pro-atherogenic manner. The responses in mice were closer to the ones in healthy men. This study presents striking differences in postprandial triglycerides patterns between rats and mice not correlated to baseline triglycerides, the animal baseline body weight or fat load in all animal groups.

## Background

The postprandial lipid metabolism has pro-atherogenic characteristics and includes a series of metabolic events that occur following the ingestion of a fat rich meal [1-4]. In normolipidemic individuals, the duration of the process is extended to 4 to 6 hours with an absorption triglyceride (TG) peak at 2 h [3], and in individuals with alteration in the lipid profile, the period can be larger than 6 hours [3]. Postprandial lipemia can be measured traditionally through two conventional methods: either by ingestion of fats (oral fat tolerance test) [3] or the intravenous administration of radio labeled lipids [5].

The pathophysiology of postprandial lipemia is not yet entirely clarified and possibly the response to dietary fat is a polygenic phenomenon [6]. It is suggested that the limiting fat intake to approximately 30 g on each eating occasion would minimize postprandial lipemia [1].

Although many studies have shown postprandial lipemia in humans [7], in animal models such as rats and mice, largely used to study the mechanisms of atherogenesis, there is a lack of consistent information on the behavior after an oral fat challenge.

Previous studies in our laboratory additionally have described postprandial lipemia in humans with very peculiar differences between sexes [8].

The objective of the present study was to characterize and compare the post-alimentary TG profile of two members of the Muridae family, the Wistar rats (Rattus norvegicus albinus) and C57BL/6 mice (Mus musculus) and compare them to the well described profile obtained in humans.

## Methods

Thirty-one male, twelve female Wistar (w) rats and ten C57BL/6 male and nine female mice (c), were obtained from the Multidisciplinary Center of Biological Investigation (CEMIB), State University of Campinas (UNICAMP), all 8 weeks old, weighing on average 190 ± 6 g (w) and 24 ± 0.7 g (c), respectively, were used in this study. The animals were housed (4 animals per cage) in a temperature-controlled room (22°C) with 12-h light-dark cycles and received a standard rodent diet (Nuvital, Curitiba, PR, Brazil) and water ad libitum. All animals underwent an oral fat tolerance test. They were lightly anesthetized with ether for tail blood collection after a 12 h fast and after ingestion of a milk cream (Batavo^®^, Brazil) by gavage. Serial blood samples were collected at 2, 4, 6, 8 and 10 h after the ingestion in rats and 1, 2, 3, 4, 5 and 6 h in mice. The liquid meal contained fat (17%), protein (4%) and carbohydrates (4%), providing 2 g fat/Kg (w and c).

In humans, the test began by venous puncture after a 12-h fast followed by ingestion of a milkshake prepared with lactose-free powdered milk (NAN, Nestlé, São Paulo, Brazil). The liquid meal contained fat (25%), dextromaltose (55%), protein (14%), and vitamins and minerals (6%), providing 40 g of fat per square meter of body surface, and was given over a period up to 10 min. Serial blood samples were collected at 2, 4, 6, and 8 h after ingestion.

Measurements were performed on samples from all time points or from the TG peak and/or 8 h. Serum TG were determined by a standard enzymatic method (GK/GPO/Roche) in an automated system (humans) or using a manual microplate method (animals). The areas under the incremental curves (AUIC) were calculated by the trapezoidal rules and the acquisition and removal rates (AR and RR) were calculated as the slopes of the curves from 0 to peak time and from peak time to 0 h, respectively. The TG peaks were classified in early peaks (high value until 4 h) and late peaks (high value after 4 h).

The experimental protocols for rodents and humans were respectively approved by the Ethics Committee for Animal Experimentation (Biology Institute, State University of Campinas, Campinas, SP, Brazil) and by the Ethics Committee of the School of Medicine of the State University of Campinas, São Paulo.

## Results

**Figure 1:**
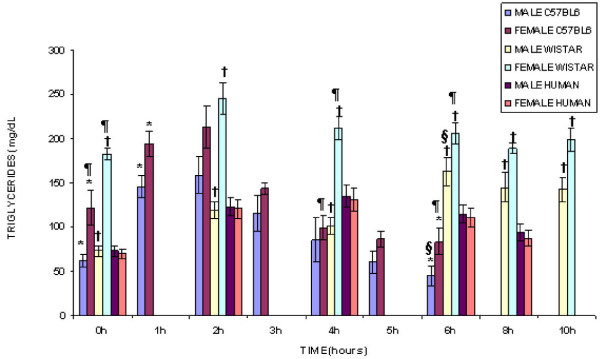
**Postprandial Triglyceridemia in Rats, Mice and Humans**. Data as means ± SEM; Mann-Whitney tests: *males and females: C57BL/6 mice (p < 0.03); ^† ^males and females: Wistar rats (p < 0.002); ^§ ^male rats and male mice (p ≤ 0.001); ^¶^differences between female rats and female mice (p ≤ 0.01).

Figure 1 shows the postprandial triglyceridemia in Wistar rats, in C57BL/6 mice and in humans.

Male and female Wistar rats showed differences in TG at all times, while male and female C57BL/6 mice only showed differences at 0, 1 and 6 hours after ingestion of milky cream. The frequency of late TG peak was significantly higher (p < 0.002) in male Wistar (84%) as compared to the other groups (female Wistar 16%, both sexes C57BL/6 did not show late peak).

When male Wistar rats and male C57BL/6 mice were compared, there was a difference at 6 h only. However, the female Wistar rats and C57BL/6 mice, were different at 0, 4 and 6 h.

Table 1 presents the postprandial TG responses as the percentage of baseline variation, area under curves and acquisition/removal rates in Wistar rats, C57/BL6 mice and human.

**Table 1:** Postprandial Triglycerides Responses in Wistar Rats, C57/Bl6 Mice, Men and Women

Groups/Postprandial data	Percentage of baseline (0 h) variation								Areas Under curves (mg/dLh)		Acquisition/removal rates (mg/d/h)	
	**1 h**	**2 h**	**3 h**	**4 h**	**5 h**	**6 h**	**8 h**	**10 h**	**AUC TG**	**AUIC TG**	**AR TG**	**RR TG**
	
**Wistar Male (31)**	-	**86 **^**a**^	-	**70 **^**a**^	-	**158 **^**a**^	**127 **^**a**^	**125 **^**a**^	**1269 ± 105**^**g**^	**539 ± 97**^**h**^	18 ± 3	**21 ± 3**^**l**^
**Wistar Female (12)**	-	**35 **^**b**^	-	**19 **^**b**^	-	**17 **^**b**^	11	15	**2085 ± 102**^**g**^	**257 ± 91**^**h**^	30 ± 8	**201 ± 21**^**l**^
**CL57BL/6 Male (10)**	**59 **^**c**^	**96 **^**c**^	**118 **^**c**^	105	54	76	-	-	**619 ± 84**^**i**^	249 ± 92	68 ± 10	50 ± 14
**CL57BL/6 Female (9)**	**91 **^**d**^	**108 **^**d**^	51	30	31	38	-	-	**839 ± 54**^**i**^	107 ± 88	86 ± 15	46 ± 6
**Men (31)**	-	**79**^**e**^	-	**87**^**e**^	-	**66**^**e**^	**32**^**e**^	-	**1051 ± 74**^**j**^	**402 ± 46**^**k**^	24 ± 3	19 ± 3
**Women (29)**	-	**57**^**f**^	-	**78**^**f**^	-	**44**^**f**^	**26**^**f**^	-	**734 ± 46**^**j**^	**238 ± 29**^**k**^	16 ± 2	12 ± 2

Data as means ± SEM; Groups (n); Diet chow intake: 15 g/day; water intake: 30 mL/day. weight of rats: 190 ± 6 g and mice: 24 ± 0.7 g; Percentage of baseline (0 h) variation, areas under curves (AUC) and incremental areas under curves (AUIC); Wilcoxon test: a, b, c and d: differences from 0 h. a < 0.000; b < 0.03, c < 0.008, d < 0.02, e < 0.001, f < 0.003. Mann-Whitney test; g < 0.0001, h < 0.04, i < 0.003, j < 0.002, k < 0.004, l < 0.0001.

## Discussion

The present study presents striking differences in postprandial TG patterns between rats and mice and by sexes. The TG responses to the meal were not correlated to the animal baseline body weight or fat load in all animal groups or baseline TG. The responses in mice were closer to the ones in humans.

All the groups have shown differences in AUC TG, while differences in AUIC were only found in the Wistar group and in humans.

The female Wistar group has shown a bigger removal rate when compared to the males.

It is evident that postprandial lipemia may contribute to increased progression of CVD (cardiovascular disease). TG-rich lipoproteins are involved in atherosclerosis and thrombosis. TG, remnant-like particle (RLP)-cholesterol (C) and RLP-TG increase after a fat load and could contribute to endothelial dysfunction and atherothrombosis [6,9]. Humans and animals in western civilizations are potentially in a continuous postprandial state for up to 16-20 h per day [10].

The rat has been a mainstay of physiological and metabolic research since the development of the first defined rat strain at the Wistar Institute in the 1920s [11].

The normal rat, like the dog, is typically resistant to the development of atherosclerosis, which has led to the notion that the rat is not a suitable subject for cardiovascular research. This idea is correct regarding the "normal" rat, but is not necessarily correct in the case of genetic models of cardiovascular disease (CVD) that have been developed over recent years. The situation is similar on the human population, where some individuals are at high risk for CVD and others are highly resistant, and the "normal" rats provide a valuable negative control in experimental studies [12].

The lipid metabolism of the "normal" rat and mice is primarily based on HDL, rather than on LDL as in humans, which certainly contributes to their resistance to atherosclerosis [12,13].

We demonstrated that mice and female rats presented TG responses similar to the ones observed in men [8] and were more fat resistant than male rats. As well, late TG responses were observed in rats as opposed to mice shown by a 5 fold higher late TG peak frequency in rats. Thus, after an oral fat load, rats are prone to respond in a pro-atherogenic manner by either facilitating the saturation of lipolytic pathways, reducing the clearance capacity of cholesterol-rich lipoproteins, or exacerbating the permeability and retention of cholesterol in arterial vessels [12,14]. Besides, male rats were fat intolerant as compared to female rats. Differently, mice TG responses were equal in both sexes.

A few other studies in the literature [15] showed the peak of TG in the second hour after the administration of 10 mL/Kg of lipid emulsion composed of 200 g/L of soybean oil, 12 g/L of egg yolk lecithin, and 22,5 g/L of glycerol and given through gavage.

In a study on drugs that improve the gastric emptying in diabetic, Wistar rats were used as controls. The fat tolerance test was performed with olive oil orally administered at 10 ml/kg, and the TG peak was also in the second hour [16].

Differences in the absorption patterns due to the quality and the amount of supplied fat and different experimental methodologies applied may explain these discrepancies, but the mechanisms that regulate the divergent responses need to be better understood.

The divergences above between responses by sexes and species observed in this study will be object of subsequent studies in our laboratory, as well as the implications of these different postprandial lipemia patterns on resistance to endothelial dysfunction and atherosclerosis.

Besides this researchers should be aware of the postprandial lipemia and always implement fasting in metabolic experiments with animals.

## Abbreviations

AR: acquisition rate; RR: removal rate; AUC: area under the curve; AUIC: area under the incremental curve; CVD: cardiovascular disease; HDL: high-density lipoprotein; LDL: low-density lipoprotein; RLP: remnant-like particle; C: cholesterol; TG: triglycerides

## Competing interests

The authors declare that they have no competing interests.

## Authors' contributions

NBP updated the references, worked hard in the data analysis, wrote parts of the manuscript, corrected it, reviewed it cautiously until the final version. AU discussed the design of the study, participated actively in the collection of data and wrote the first draft of the manuscript. ESP reviewed the manuscript critically, edited it and submitted it to publication. RO was the responsible for the statistical analyses. VSZ reviewed the English several times until its final edition. LRS observed the data analysis and participated in the interpretation of the results. ECF coordinated this work at all times, created the design of the study, and implemented it in the clinical yard and in. She also analyzed the methods, results, and statistical data and prepared the final manuscript. All authors read and approved the manuscript.

## Aknowledgements

This study was developed in part by grants from the Fundação de Amparo à Pesquisa do Estado de São Paulo (FAPESP) and Conselho Nacional para o Desenvolvimento Científico e Tecnológico (CNPq).
